# Long-term outcomes of fully covered self-expandable metal stents versus plastic stents in chronic pancreatitis

**DOI:** 10.1038/s41598-021-94726-z

**Published:** 2021-08-02

**Authors:** Sang Hoon Lee, Yeon Suk Kim, Eui Joo Kim, Hee Seung Lee, Jeong Youp Park, Seung Woo Park, Si Young Song, Jae Hee Cho, Seungmin Bang

**Affiliations:** 1 Department of Internal Medicine, Severance Hospital, Yonsei University College of Medicine, 50 Yonsei-ro, Seodaemun-gu, Seoul, 03772 Republic of Korea; 2 Department of Internal Medicine, Konkuk University School of Medicine, Seoul, South Korea; 3 Department of Internal Medicine, Gachon University Gil Medical Center, Gachon University College of Medicine, Incheon, Republic of Korea; 4 Department of Internal Medicine, Gangnam Severance Hospital, Yonsei University College of Medicine, 211 Eonju-ro, Gangnam-gu, Seoul, 06273 Republic of Korea

**Keywords:** Chronic pancreatitis, Chronic pancreatitis

## Abstract

Chronic pancreatitis (CP) related main pancreatic duct (MPD) stricture has been a challenge for endoscopists. Fully covered self-expandable metal stents (FC-SEMS) has been tried in CP patients, but the efficacy and safety are still controversial. Thus, we aim to compare the long-term clinical efficacy of FC-SEMS vs. plastic stent placement in persistent MPD strictures secondary to CP. Between 2007 and 2018, 80 chronic pancreatitis patients (58 males, median age 49 years), who underwent endoscopic placement of FC-SEMS (n = 26) and plastic stent (n = 54) for persistent MPD strictures after at least 3 months of initial single plastic stenting, were retrospectively analyzed during a median follow-up duration of 33.7 months. As a result, MPD stricture resolution rate was statistically higher in FC-SEMS group (87.0% vs. 42.0%, *p* < 0.001). Although immediate complications occurred similarly (38.5% vs. 37.0%, *p* = 0.902), spontaneous migration (26.9%) and de novo strictures (23.1%) were pronounced delayed complications in FC-SEMS group. Pain relief during follow-up was significantly higher in FC-SEMS group (76.9% vs. 53.7%, *p* = 0.046). The total procedure cost was similar in both groups ($1,455.6 vs. $1,596.9, *p* = 0.486). In comparison with plastic stent, FC-SEMS placement for persistent MPD strictures had favorable long-term clinical efficacy, with its typical complications like spontaneous migration and de novo strictures.

## Introduction

Chronic pancreatitis (CP) is a benign, irreversible, inflammatory pancreatic disorder, which can result in various complications including symptomatic main pancreatic duct (MPD) stricture. In fact, MPD stricture occurred in up to 47% of CP patients who received endoscopic treatment^1^. Endoscopic pancreatic duct (PD) stenting with a single plastic stent has been mainly used for treatment of CP patients with symptomatic MPD strictures^2–4^. However, PD stents cannot be completely removed in approximately one-third of patients because of persistent or recurrent strictures^1,4–6^. Therefore, patients with unrelenting symptomatic MPD strictures may require regular plastic stent exchanges indefinitely. Although surgical intervention is a definitive treatment option with long-term pain relief, patients often refuse it because of its invasiveness and associated adverse events^7^. Persistent MPD strictures after initial plastic stent placement has been a challenge for endoscopists.

Fully covered self-expandable metal stent (FC-SEMS) placement was lately introduced as an investigated treatment option for persistent MPD strictures after single plastic stenting^3^. FC-SEMS has advantages including a larger diameter, longer patency duration, and technical ease to release compared with plastic stent^8,9^. Several published data have recently demonstrated clinical efficacy of FC-SEMS as a 37–88% pain improvement during a 3–4 year follow-up period^10–12^. However, these pilot studies occurred at a single medical center, and were limited by a small sample size (10–18 patients). To date, no studies have compared FC-SEMS and plastic stent placement for symptomatic CP patients with benign MPD strictures^13^. Therefore, we aimed to compare the long-term clinical efficacy of FC-SEMS vs. plastic stent placement in CP patients with persistent MPD strictures after at least 3 months of single plastic stenting in two medical institutions.

## Results

### Patients’ characteristics and stent feature

Baseline characteristics and stent feature of FC-SEMS and plastic stent group were summarized in Table 1. Median age was 49 years old and 58 (72.5%) patients were male. The majority of the MPD strictures were located in the pancreas head (n = 62, 77.5%). Other complications of CP such as PD stones, pseudocysts, and biliary strictures occurred simultaneously in both groups from the beginning, but their incidences were not significantly different between groups. During the median 24.9 and 36.2 months of follow-up, procedures related to these complications were excluded in following analyses.

**Table 1:** Comparison of patients’ characteristics and stent feature.

	FC-SEMS group (n = 26)		Plastic stent group (n = 54)		P -value
Age, median (IQR), years	47.0 (40.3—49.3)		57.0 (38.0—65.3)		0.075
Male, No. (%)	19 (73.1)		39 (72.2)		0.936
**Etiology**					
Alcohol/Hereditary/Idiopathic, No. (%)	20 (76.9)/ 4 (15.4)/ 2 (7.7)		35 (64.8)/ 5 (9.3)/ 14 (25.9)		0.144
Location of stricture					
Head/Neck, No. (%)	23 (88.5)/ 3 (11.5)		39 (72.7)/ 15 (27.8)		0.103
**Combined complication**					
Pancreatic duct stones, No. (%)	15 (57.7)		22 (40.7)		0.154
Pseudocyst, No. (%)	5 (19.2)		14 (25.9)		0.510
Biliary stricture at distal CBD, No. (%)	4 (15.4)		6 (11.1)		0.588
Follow-up duration, median (IQR), months	24.9 (11.4—57.7)		36.2 (12.7—85.6)		0.237
**Stent feature**					
Diameter^¶^, No. (%)	8 mm	12 (46.2)	5Fr	12 (22.2)	
	10 mm	14 (53.8)	7Fr	26 (48.1)	
			8.5Fr	2 (3.7)	
			10Fr	14 (25.9)*	
Length, cm	4 ~ 8		5 ~ 12		

^¶^For the plastic stent group, the maximum stent diameters were described.

*Including 4 patients with multiple plastic stent placement (n = 3, 7Fr + 7Fr; n = 1, 10Fr + 7Fr).

Abbreviations: FC-SEMS, fully covered self-expandable metal stent; PS, plastic stent; IQR, interquartile range; CBD, common bile duct.

For the plastic stent group, the maximum stent diameters were described in Table 1. In fact, we failed to upsize the plastic stent diameter in 19 patients (35.2%) in the plastic stent group. The most frequently used length was 6 cm (n = 9, 34.6%) for FC-SEMS, and 5 cm (n = 18, 33.3%) and 7 cm (n = 17, 31.5%) for plastic stents. The types of FC-SEMSs and plastic stents are listed in Supplementary Tables 1 and 2.

### Procedure-related outcomes

Procedure-related outcomes are summarized in Table 2. Technical success was achieved in all patients of both groups (100%). The total duration of stent placement was significantly shorter in the FC-SEMS group than in the plastic stent group (median 4.9 vs. 7.3 months, *p* = 0.022). When stent placement duration was calculated from the date of initial plastic stenting, not from the index procedure date, the median duration for plastic stenting was 10.5 (interquartile range [IQR], 6.0–21.9) months in the plastic group.

**Table 2:** Comparison of procedure-related outcomes.

	FC-SEMS group (n = 26)	Plastic stent group (n = 54)	p-value
Technical success, No (%)	26 (100)	54 (100)	> 0.99
Stent exchange, No (%)	1 (3.8)	25 (46.3)	< 0.001
Exchange interval, median (IQR), months	3.0	6.1 (3.4–7.8)	
Stent placement duration, median (IQR), months	4.9 (4.0—6.5)	7.3 (3.7—15.2)	0.022
Stent removal, No (%)	24 (92.3)	37 (68.5)	0.019
Spontaneous migration, No (%)	7 (26.9)	2 (3.7)	0.002
Scheduled removal, No (%)	17 (65.4)	35 (64.8)	0.960
MPD stricture resolution, No (%)	20 (87.0) ^¶^	21 (42.0) ^¶¶^	< 0.001
Immediate complications, No (%)	10 (38.5)	20 (37.0)	0.902
Cholangitis, No (%)	1 (3.8)	2 (3.7)	0.975
Pancreatitis, No (%)	5 (19.2)	11 (20.4)	0.905
Hyperamylasemia, No (%)	6 (23.1)	8 (14.8)	0.362
Perforation, No (%)	0 (0)	1 (1.9)	0.485
**Delayed complications**			
Proximal migration, No (%)	2 (7.7)	1 (1.9)	0.198
Stent malfunction, No (%)	0 (0.0)	2 (3.7)	0.320
Stent fracture, No (%)	2 (7.7)	–	0.039
De novo MPD stricture, No (%)	6 (23.1)	–	< 0.001

^¶^ Excluding 3 patients without follow-up pancreatography after stent removal.

^¶¶^ Excluding 4 patients without follow-up pancreatography after stent removal.

Abbreviations: FC-SEMS, fully covered self-expandable metal stent; IQR, interquartile range; MPD, main pancreatic duct.

**Figure 1:**
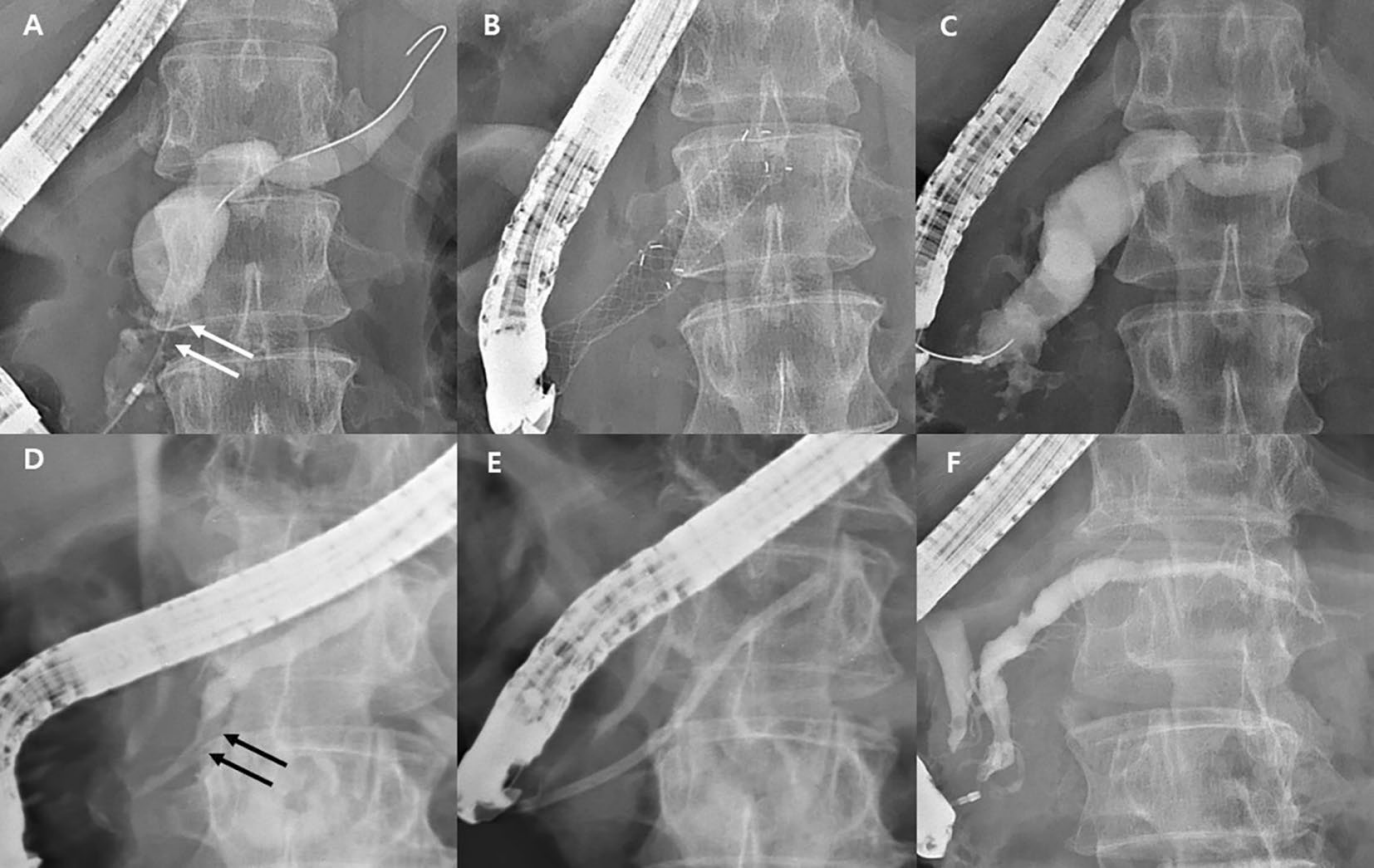
Fluoroscopic images of endoscopic retrograde pancreatography. Pancreatography shows a pancreatic ductal stricture in the head (white arrows) with upstream duct dilatation (**a**). After 7 months of FC-SEMS placement (**b**), follow-up pancreatography reveals stricture resolution (**c**). Pancreatography shows a tight pancreatic duct stricture in the pancreas head (black arrows) with upstream duct dilatation (**d**). After 13 months of single plastic stent placement (**e**), follow-up pancreatography reveals stricture resolution (**f**). Abbreviation: FC-SEMS, fully covered self-expandable metal stent.

In the FC-SEMS group, almost all patients (n = 24, 92.3%) removed the stent, with the exception of two patients who retained the FC-SEMS until the last follow-up (their FC-SEMS placement duration were 0.4 and 2.9 months). However, 31.5% of patients in the plastic stent group continued with MPD stenting (*p* = 0.019). MPD stricture resolution was statistically higher in FC-SEMS group (87.0% vs. 42.0%, *p* < 0.001). (Fig. 1).

Immediate complications after stent insertion occurred similarly in both groups (38.5% vs. 37.0%, *p* = 0.902). One-fourth of the FC-SEMS group developed spontaneous migration with median 3.1 months interval after stent insertion, which was more dominant than in the plastic stent group patients (26.9% vs. 3.7%, *p* = 0.002). In addition, other delayed complications such as stent fracture (7.7%) and de novo stricture (23.1%) occurred frequently in the FC-SEMS group (*p* = 0.039 and < 0.001, respectively). Of the six patients who developed de novo strictures after FC-SEMS removal, five were asymptomatic while one was clinically manageable with temporary plastic stenting for 3.8 months.

### Clinical outcomes

**Figure 2:**
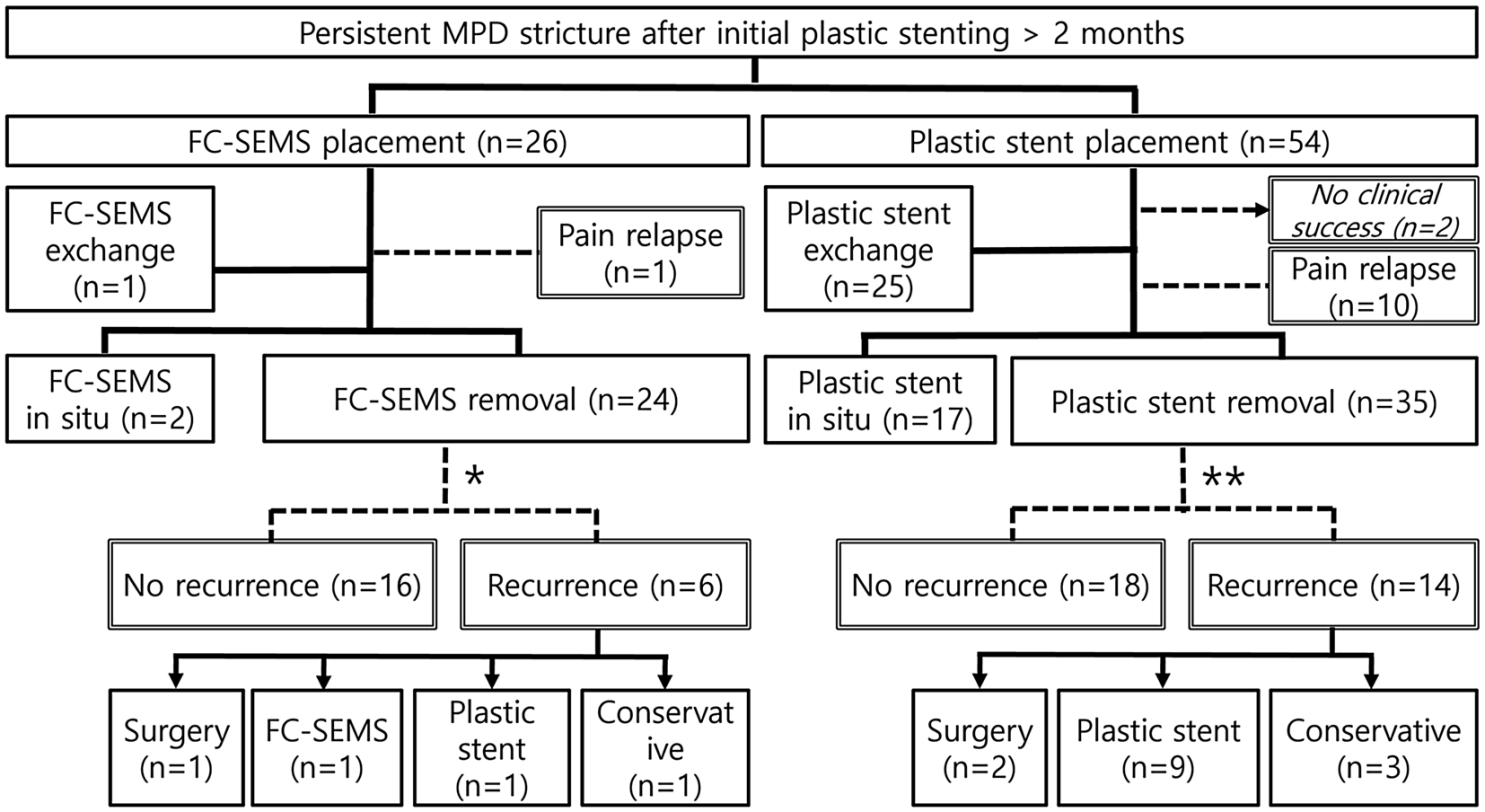
Flowchart for clinical outcomes. Exclusion of patients with failure of additional stenting of remained stricture (*n = 2; **n = 3). Abbreviations: FC-SEMS, fully covered self-expandable metal stent; MPD, main pancreatic duct.

Clinical outcomes in both groups are shown in Fig. 2 and Table 3. Two patients without clinical success in the plastic stent group were excluded in following analyses. Compared to the FC-SEMS group, about 20% of the plastic stent group suffered significantly from pain relapse during stent placement (3.8% vs. 19.2%, *p* = 0.066).

**Table 3:** Comparison of clinical outcomes.

	FC-SEMS group (n = 26)	Plastic stent group (n = 54)	p-value
Clinical success, No (%)	26 (100)	52 (96.3)	0.320
Pain relapse during stent placement, No (%)	1 (3.8)	10 (19.2)†	0.066
Pain relapse interval, median (95% CI), months	2.6	4.1 (1.0—7.3)	0.384
Recurrence, No (%)	6 (27.3) ^¶^	14 (43.8) ^¶¶^	0.587
Recurrence-free survival, median (95% CI), months	50.1 (31.2- 69.1)	51.7 (28.7 -74.7)	0.646
Pain relief during follow-up, No (%)	20 (76.9)	29 (53.7)	0.046

^†^ Excluding 2 patients without clinical success.

^¶^ Excluding 2 patients with failure of additional stenting for remained stricture.

^¶¶^ Excluding 3 patients with failure of additional stenting for remained stricture.

Abbreviations: FC-SEMS, fully covered self-expandable metal stent; CI, Confidence interval.

**Figure 3:**
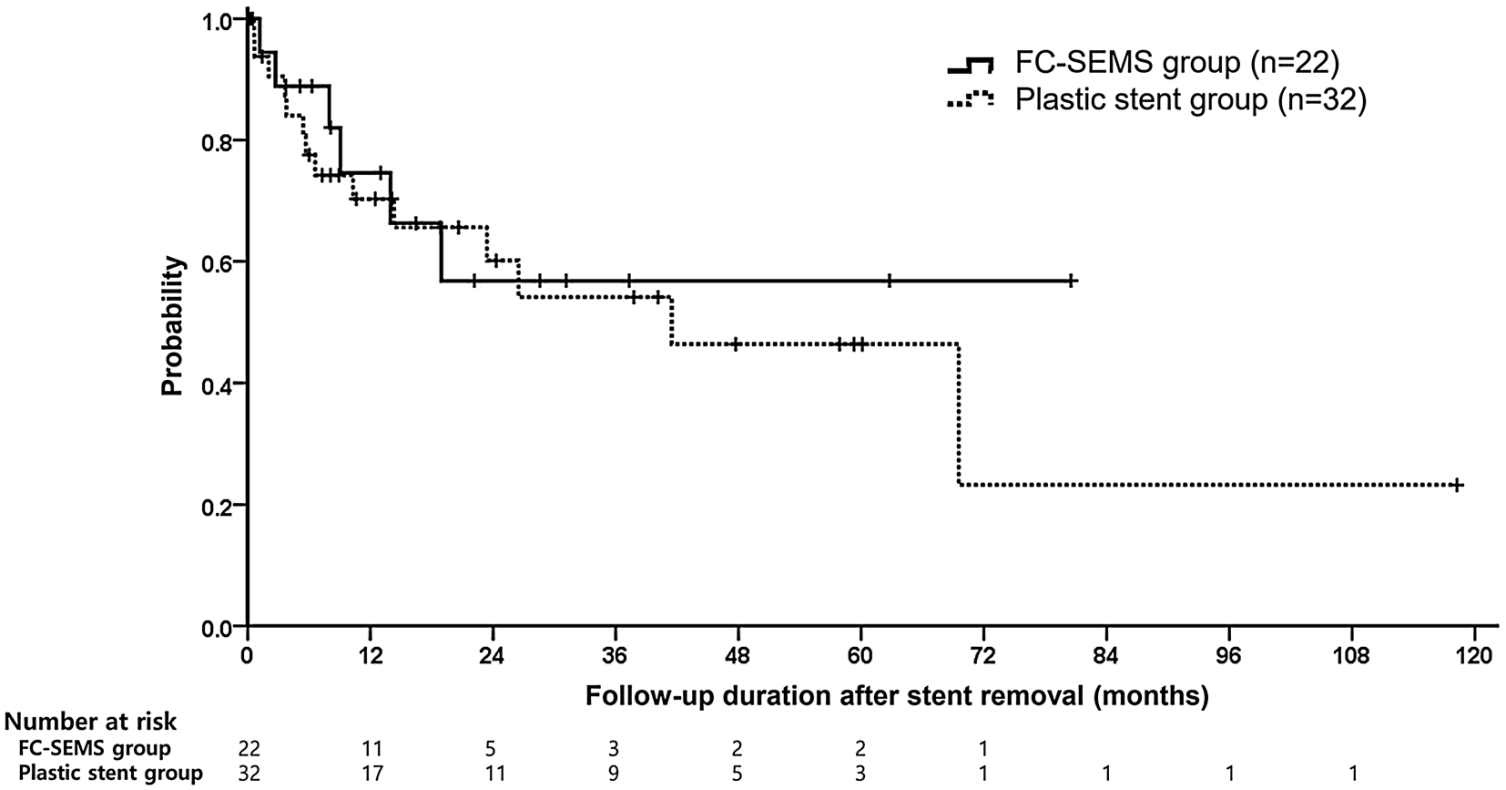
Kaplan-Meir estimation of recurrence-free survival after stent removal in the FC-SEMS (n = 22) and plastic stent group (n = 32). There were no significant differences in recurrence-free survival between the FC-SEMS and plastic stent groups (50.1 (95% CI, 31.2—69.1) vs. 51.7 (95% CI, 28.7—74.7) months, log-rank p = 0.646). Abbreviation: FC-SEMS, fully covered self-expandable metal stent; CI, confidence interval.

The proportion of patients who showed recurrence after stent removal and recurrence-free survival were not statistically different between the two groups (27.3% vs. 43.8%, *p* = 0.587; mean 50.1 vs. 51.7 months, *p* = 0.646, respectively) (Fig. 3). Finally, in our study population, more patients in FC-SEMS group reported experiencing pain relief during follow-up without pain relapse or recurrence (76.9% vs. 53.7%, *p* = 0.046).

### Cost-effectiveness analysis

To compare cost-effectiveness of the stents, we calculated total number of endoscopic retrograde cholangiopancreatography (ERCP) sessions and summed the total cost of procedures (Table 4). Total number of procedures was significantly lower in the FC-SEMS group (median 2 vs. 3, *p* = 0.014), but the mean cost between the two groups ($1,455.6 vs. $1,596.9, *p* = 0.486) was not different.

**Table 4:** Comparison of number of procedure and cost analysis.

	FC-SEMS group (n = 26)	Plastic stent group (n = 54)	p-value
Number of ERCP, median (range)	2 (1 ~ 3)	3 (1 ~ 10)	0.014
Total cost, mean (SD), US$	1455.6 (333.1)	1596.9 (1000.8)	0.486

Abbreviations: FC-SEMS, fully covered self-expandable metal stent; ERCP, endoscopic retrograde cholangiopancreatography.

## Discussion

This was the first study to describe and compare the long-term clinical efficacies of FC-SEMS and plastic stent placements in a clinical setting to treat persistent MPD strictures in patients with CP. Although FC-SEMS showed typical delayed complications, such as de novo stricture and spontaneous migration, its clinical efficacy showed a higher MPD stricture resolution rate and considerable sustained pain relief during follow-up.

Compared to a plastic stent, FC-SEMS has a larger diameter and longer patency. Further, it is technically easier to release FC-SEMS than even a single plastic stent owing to its superior “pushability,” with higher flexibility and a lower external diameter of the pushing catheter (7 vs. 10 Fr)^8,9^. Technically, all FC-SEMSs were successfully inserted and removed, including three stent fracture cases and two proximal migration cases. Furthermore, the larger diameter of FC-SEMS could help improve the MPD stricture. According to previous studies, stricture resolution was achieved in 83–100% of patients treated with FC-SEMS^9–12,14^ and in 9–50% of patients with single plastic stenting^5,15–18^.

Temporary placement of FC-SEMS, together with surgery and multiple side-by-side plastic stent placement, is currently considered to be a treatment option for refractory MPD strictures, which are defined as a symptomatic dominant stricture persisting or relapsing after 1 year of single plastic stenting^3^. Comparing clinical data of FC-SEMS placement for symptomatic MPD strictures in patients with CP (Supplementary Table 3) has several complicated and discordant issues. First, clinical indication for FC-SEMS placement varies. Some early studies enrolled all symptomatic patients with an MPD stricture, independent of plastic stent placement before FC-SEMS insertion^9,14^. In contrast, other studies, including ours, included only patients with persistent or refractory stricture with inclusion criteria of a 3—^10,12^ or 12-month^11^ duration of previous plastic stent placement. When the FC-SEMS group was divided into subgroups before and after a plastic stent indwelling period of 6 months in our results, the FC-SEMS group under 6 months of plastic stent indwelling (n = 13, 50.0%) showed comparable clinical efficacy of MPD stricture resolution, recurrence rates, and pain relief during follow-up (91.7% vs. 81.8%, p = 0.484; 80.0% vs. 66.7%, p = 0.484; 84.6% vs. 69.2%, p = 0.352, respectively) compared to the remainder subgroup (n = 13, 50.0%). Because evidence for FC-SEMS is currently lacking, further clinical trials are required for the indications and timing of FC-SEMS placement in patients with CP.

Insertion of a single 10-Fr plastic stent for 1 year was recommended as the initial endoscopic therapy for MPD strictures^3,19,20^. However, achieving the maximum calibration of plastic stenting can be technically difficult because of the tightness of the MPD stricture. In our study, the diameter of the plastic stent could not be increased in 19 (35.2%) patients, and approximately three-quarters (74.1%) of patients retained a single plastic stent with a diameter < 8.5 Fr until the last follow-up (Table 1). When we divided the plastic group based on the maximum diameter of the plastic stent, the recurrence rate or recurrence-free survival showed no significant difference among subgroups (Supplementary Table 4).

Notably, spontaneous migration developed in one-fourth (26.9%) of patients in the FC-SEMS group, and other delayed complications, such as stent fracture (7.7%) and de novo stricture (23.1%), were also prevalent (*p* = 0.002, 0.039, and < 0.001, respectively). Reported frequencies of stent migration and de novo strictures for FC-SEMS are 15–46% and 16–27%, respectively^10,12,14,21^ (Supplementary Table 3). Tringali et al. reported that the short 3-cm FC-SEMS may be associated with frequent stent migrations^12^, and Oh et al. suggested that additional anchoring inner plastic stents help prevent stent migration^11^. Anti-migration systems have recently been developed and applied to FC-SEMS^21,22^, but some concerns exist because of its flared end, which may cause stent-induced de novo stricture^10,12,14,21^.

Our study has several limitations. First, this was a non-randomized retrospective study. Although statistically not significant in baseline characteristics, alcohol consumption and other CP complications during follow-up may affect the pain aggravation event. In addition, stent features of FC-SEMS and plastic stents were not unified, and there was no consistent strategy for choosing FC-SEMS or plastic stents. Some patients treated with plastic stents sub-optimally showed less effective clinical outcomes in the plastic stent group. Data maturity issues make it difficult to estimate meaningful recurrence-free survival. Finally, we analyzed cost-effectiveness simply resulting from our relatively small study population, excluding the length of hospitalization and complication rates.

In conclusion, FC-SEMS placement for persistent MPD stricture in patients with CP had favorable long-term clinical efficacy with a higher MPD stricture resolution rate and sustained pain relief during follow-up compared to plastic stenting. Typical complications of FC-SEMS, such as de novo stricture and spontaneous migration, remain major issues, necessitating further investigations.

## Methods

### Study population

**Figure 4:**
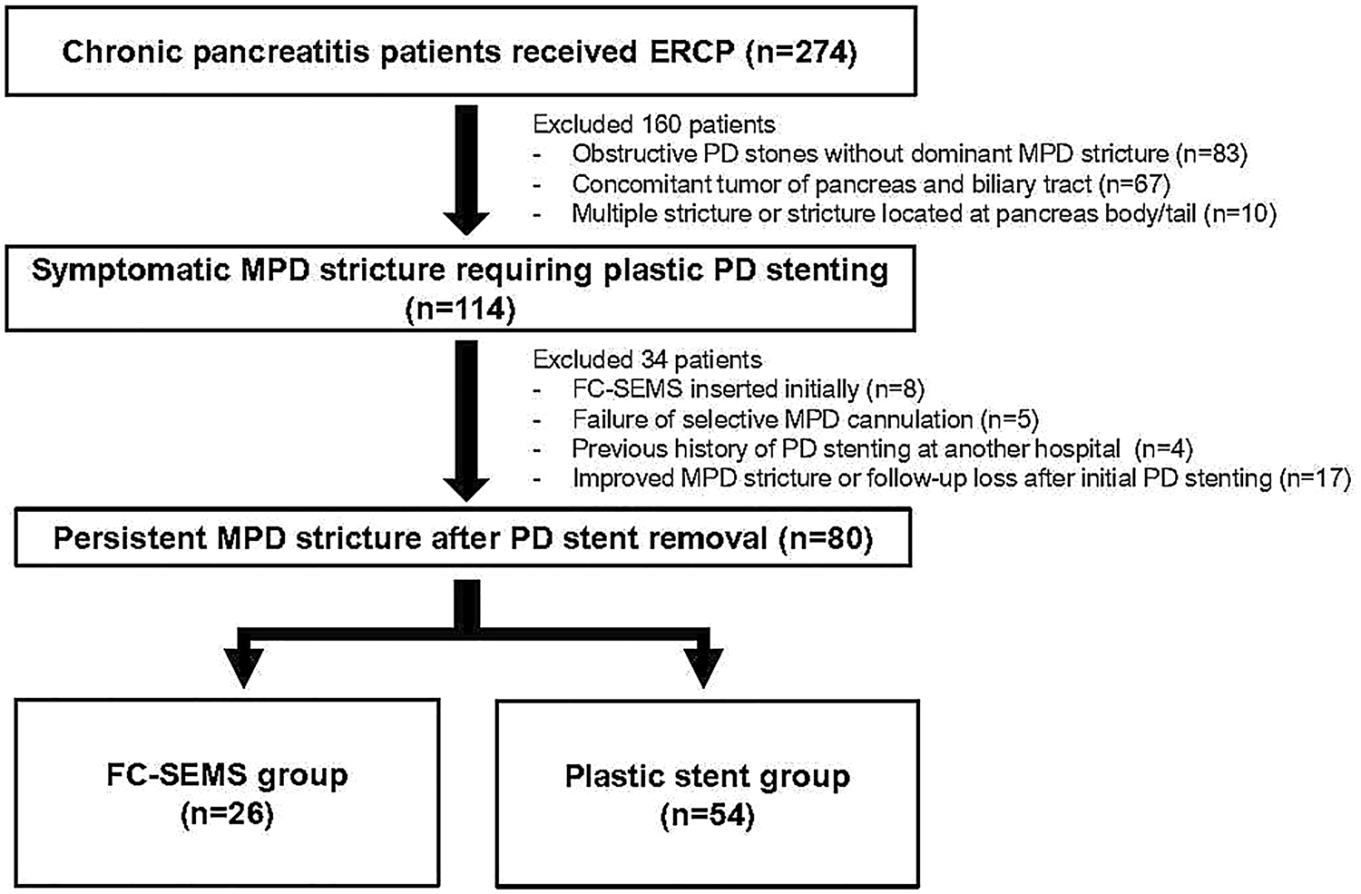
Flowchart for the study population. Abbreviation: ERCP, endoscopic retrograde cholangiopancreatography; FC-SEMS, fully covered self-expandable metal stent; MPD, main pancreatic duct; PD, pancreatic duct.

We retrospectively collected data from symptomatic CP patients with persistent MPD strictures after single plastic stent (5-10Fr) placement for at least 3 months in Severance hospital and Gil medical center between January 2007 and December 2018. Patients were included according to the following criteria: (1) dominant MPD stricture located in pancreatic head and neck; (2) initially treated with single plastic stent insertion; (3) persistent MPD stricture with upstream PD dilatation after initial plastic stent removal. Exclusion criteria were as follows: (1) obstructive PD stones without dominant MPD stricture; (2) concomitant tumor of pancreas and biliary tract; (3) multiple MPD strictures or strictures located at the pancreas body/tail; (4) FC-SEMS inserted initially; (5) failure of selective MPD cannulation; (6) previous history of PD stenting at another hospital; and (7) improved MPD stricture or follow-up loss after initial PD stenting. Finally, 80 CP patients with persistent MPD strictures were included in our analysis. According to types of secondary PD stents, those were divided into FC-SEMS groups (n = 26) and plastic stent group (n = 54) (Fig. 4).

The study protocol conformed to the ethical guidelines of the 1975 Helsinki Declaration and was approved by the institutional review board (IRB) of each institute (Severance Hospital IRB, 4-2019-2196; Gachon University Gil Medical Center IRB, GDIRB2019-410). The need for informed consent was waived by ‘Severance Hospital Institutional Review Board’ and ‘Gachon University Gil Medical Center Institutional Review Board’ due to the retrospective study design.

### Procedures

Experienced endoscopists examined ERCP. When MPD stricture was persistent on subsequent pancreatography after removal of the first inserted plastic stent, the physician would try to insert a single plastic stent with a larger diameter, perform multiple plastic stent stenting, or use a FC-SEMS. If advancing the catheter or introducing the plastic stent and SEMS through the stricture was difficult, a dilating catheter (6–8.5 Fr Soehendra dilatation catheter, Cook Medical) and/or balloon dilator (4–6 mm Hurricane Rx balloon dilators, Boston Scientific) were/was used. The stent diameter was chosen according to stricture tightness and diameter of the upstream-dilated MPD. Stent length was decided based on stricture location and MPD morphology.

Stents were exchanged or removed every 3–6 months after placement. When patient’s pain relieved and MPD stricture was disappeared on follow-up pancreatography, stents were removed. Especially in cases of FC-SEMS insertion, we did not maintain the FC-SEMS placement for longer than six months. Therefore, if it was necessary to insert an additional stent for a remaining stricture or de novo stricture after 6 months of FC-SEMS placement, we used a plastic stent.

### Follow-up

Outpatient follow-up was scheduled at 1, 3, and 6 months after stent insertion or exchange. After stent removal, follow-up was continued at 6-month intervals or whenever complications occurred. Physicians and registered nurses carefully assessed the pain intensity before stent insertion at an outpatient clinic visit, daily during hospitalization for procedure, and at each scheduled follow-up outpatient visit. ERCP was considered when the pain was aggravated or when procedure-related complications developed. The last follow-up was performed in June 2019.

### Study endpoints

When analyzing procedure-related outcomes, we regarded the second ERCP after initial plastic stent removal as an index procedure. Technical success was defined as successful placement of the stents, FC-SEMS or plastic stent during index procedures. MPD stricture resolution was defined as a resolved stricture with complete runoff of contrast material on the follow-up pancreatography. We also analyzed the immediate and delayed complications such as proximal or distal migration, stent fracture, and stent-induced de novo stricture. Spontaneous migration was defined as radiological or endoscopic evidence of stent disappearance due to distal movement away from the stricture. A de novo stricture could develop above the intraductal end of the FC-SEMS. Other complications related to ERCP were defined according to the American Society for Gastrointestinal Endoscopy workshop^25^.

We evaluated clinical outcomes, which were entirely related to patients’ symptoms, in three periods: clinical success during index procedure, pain relapse during stent placement, and recurrence after stent removal. Clinical success was defined as a > 50% reduction in the visual analog scale (VAS) scale pain score before and after stent placement during hospitalization for the index procedure. Recurrence was defined as a > 50% elevation in the VAS scale pain score compared with that after stent removal, or pain aggravation causing hospitalization or therapeutic intervention to the MPD stricture. If such pain aggravation occurred during stent placement, we separately defined it as ‘pain relapse’, as another parameter of clinical outcomes. Finally, it means that patients with pain relief during follow-up did not experience any pain relapse or recurrence after index procedure for PD stenting.

To compare total cost of placing the FC-SEMS and plastic stent, we calculated the sum of expenses of ERCP with stent insertion, exchange, and removal, including charges for all FC-SEMS and plastic stents used in both groups after index procedure, based on an annual medical fee schedule from Korean National Health Insurance^26^. These costs were converted from Korean won to U.S. dollars according the annual average exchange rate.

### Statistical analysis

Data were expressed as the mean ± standard deviation (SD), median (IQR or range), or n (%), as appropriate. A Student’s t-test or Mann–Whitney test was used to analyze continuous variables, and a Fisher’s exact test or Pearson’s chi-square test for categorical variables. Survival rates without recurrence were estimated and compared using the Kaplan–Meier method and the log-rank test. A two-tailed P-value of < 0.05 was considered statistically significant. The statistical analysis was performed with SPSS, version 25.0 (PASW Statistics Inc., Chicago, IL, USA).

## Supplementary Information

Supplementary Information 1.

Supplementary Information 2.

Supplementary Information 3.

Supplementary Information 4.

## Data Availability

The datasets used and/or analyzed during the current study are available from the corresponding author on reasonable request.
