# Supplementary material for: Long-term outcomes of fully covered self-expandable metal stents versus plastic stents in chronic pancreatitis
Source: Sci Rep. 2021 Aug 2;11:15637. doi: 10.1038/s41598-021-94726-z (PMC8329149; doi:10.1038/s41598-021-94726-z)
Supplement: Supplementary file 1 — Supplementary Information 1. [file 41598_2021_94726_MOESM1_ESM.docx]

| **Supplementary Table 1. Types of FC-SEMS** | | |
| --- | --- | --- |
| Producer | Model | No. (%) |
| TaeWoong Medical | Niti-S^®^, D-type | 10 (38.5) |
| TaeWoong Medical | Niti-S^®^, Bumpy-type | 4 (15.4) |
| TaeWoong Medical | Niti-S^®^, ComVI-type | 2 (7.7) |
| M. I. Tech | Hanarostent^®^ | 5 (19.2) |
| S&G BioTech | EGIS, Flower^TM^ stent | 5 (19.2) |
| Abbreviations: FC-SEMS, fully covered self-expandable metal stent. | | |
