# Supplementary material for: Long-term outcomes of fully covered self-expandable metal stents versus plastic stents in chronic pancreatitis
Source: Sci Rep. 2021 Aug 2;11:15637. doi: 10.1038/s41598-021-94726-z (PMC8329149; doi:10.1038/s41598-021-94726-z)
Supplement: Supplementary file 2 — Supplementary Information 2. [file 41598_2021_94726_MOESM2_ESM.docx]

| **Supplementary Table 2. Types of plastic stent at maximum diameter** | | | | |
| --- | --- | --- | --- | --- |
| Producer | Model | Stent type | Diameter (Fr) | No. (%)* |
| COOK Medical | Geenen^®^ pancreatic stent | Straight | 5 | 12 (19.4) |
|  |  |  | 7 | 32 (51.6) |
| COOK Medical | Zimmon^®^ pancreatic stent | Single pigtail | 7 | 1 (1.6) |
| COOK Medical | Tannenbaum^®^ biliary stent | Straight | 8.5 | 1 (1.6) |
|  |  |  | 10 | 14 (22.6) |
| Boston Scientific | Advanix^TM^ biliary stent | Straight | 8.5 | 1 (1.6) |
|  |  |  | 10 | 1 (1.6) |
| * When multiple plastic stents (n=4) were calculated separately, the total number of plastic stent used was 64. | | | | |
