# Supplementary material for: Long-term outcomes of fully covered self-expandable metal stents versus plastic stents in chronic pancreatitis
Source: Sci Rep. 2021 Aug 2;11:15637. doi: 10.1038/s41598-021-94726-z (PMC8329149; doi:10.1038/s41598-021-94726-z)
Supplement: Supplementary file 3 — Supplementary Information 3. [file 41598_2021_94726_MOESM3_ESM.docx]

| **Supplementary Table 3. Comparisons of published results of pancreatic FC-SEMS insertion** | | | | | | | | | |
| --- | --- | --- | --- | --- | --- | --- | --- | --- | --- |
| Reference | No. | Diameter, range, mm | Length, range, cm | Pain relief with stent placement, % | Planned stent removal, No., months | Stricture resolution, % | Follow-up duration, months | Pain relief during follow-up, % | FC-SEMS related adverse events |
| Moon et al, 2010 | 32 | 6-10 | 4-8 | 100 | 3 | 100 | 5 (mean) | 88 | 16% de novo stricture |
| Giacino et al, 2012 | 10 | 8-10 | 4-8 | 90 | 6 | 100 | 20 (mean) | 90 | 20% cholestasis 20% SEMS impaction |
| Ogura et al, 2016 | 13 | 6 | 6-8 | 92 | 6 | 100 | 9 (median) | 85 | 15% migration 8% abdominal pain |
| Matsubara et al, 2016 | 10 | 8-10 | 5-10 | 100 | 3 | 80 | 35 (median) | 37 | 30% severe pain 25% migration 25% pancreatic ductitis 25% de novo strictures |
| Tringali et al, 2018 | 15 | 6-8 | 3-5 | 100 | 6 | 93 | 39 (median) | 89 | 46% migration 27% de novo strictures 10% cholangitis |
| Oh et al, 2018 | 18 | 6 | 5-8 | 83 | 6 | 83 | 47 (median) | 87 | 16% abdominal pain |
| Present study | 26 | 8-10 | 4-8 | 97 | 6 | 87 | 25 (median) | 77 | 27% migration 8% stent fracture 23% de novo stricture |
| Abbreviations: FC-SEMS, fully covered self-expandable metal stent. | | | | | | | | | |
