# Supplementary material for: Long-term outcomes of fully covered self-expandable metal stents versus plastic stents in chronic pancreatitis
Source: Sci Rep. 2021 Aug 2;11:15637. doi: 10.1038/s41598-021-94726-z (PMC8329149; doi:10.1038/s41598-021-94726-z)
Supplement: Supplementary file 4 — Supplementary Information 4. [file 41598_2021_94726_MOESM4_ESM.docx]

| **Supplementary Table 4. Subgroup analysis of recurrence in the plastic group according to maximum diameter of stent** | | | | | | | |
| --- | --- | --- | --- | --- | --- | --- | --- |
|  | All  (n=32) | 5 Fr  (n=8) | ≥7 Fr  (n=24)* | p-value | ≤8.5 Fr (n=24) | ≥10 Fr (n=8)* | p-value |
| Recurrence, No (%) | 14 (43.8) | 3 (37.5) | 11 (45.8) | 0.681 | 10 (41.7) | 4 (50.0) | 0.681 |
| Recurrence-free survival, median (95% CI), months | 51.7  (28.7-74.7) | Not reached | 41.5  (3.5-79.5) | 0.498 | 41.5  (8.1-74.9) | Not reached | 0.385 |
| * Including 3 patients with multiple plastic stenting (n=2, 7 Fr + 7Fr; n=1, 10Fr +7Fr) | | | | | | | |
| Abbreviations: CI, Confidence interval. | | | | | | | |
